# Buffalo Hospital of the Sisters of Charity: Surgical Department

**Published:** 1849-08

**Authors:** Franklin H. Hamilton


ART. V.—"*Buffalo Hospital of the Sisters of Charity;*" *Surgical Department. Reports of Cases of Fracture; Use of starch bandage, according to the method of M. Suetin, and a new mode of using it in cases where extension is required: also, illustrations of the value of Gutta Percha as a splint.* By DR. FRANK H. HAMILTON.

CASE 1. *Fracture of Femur, at the site of an ancient fracture.* James Leacock, aet. 30 years.—Admitted Dec. 27, 1848. Nine months before, he had fractured his femur, at the lower end of the upper third. It united in the usual time, shortened three quarters of an inch. The straight splint was used.

*Dec.* 27, he fell upon his knee with moderate force, and re-fractured the limb at the same point.

*Dec.* 27, the day of his admission, I applied my own straight splint, with lateral dressings. The limb was extended to the same length as before the last fracture occurred—shortened three quarters of an inch. On the 29th, vesications had occurred upon the top of the ankle, from the pressure of the gaiter. The extending bands were loosened, and the gaiter readjusted. Notwithstanding great care was used to prevent this event, a slough occurred above the heel, on the 3d of June. From this time to Feb. 16, we were compelled by his constant complaints, and restlessness, to change the mode of extension and support, often. The extension was made from above the ankle, from above the ham, and from above the knee, alternately, or at the same time: with adhesive plasters and cotton, or flannel rollers. Once the double inclined plane was used.

*Feb.* 16, fifty-two days from the commencement of the treatment, we found the bone united, and the limb of the same length as before.

There were present the usual sources of difficulty in this case, to which were superadded an exceedingly irritable temperament, and the previous occurrence of fracture at the same point. But we wish to call the attention, particularly, to the difficulty of making the requisite extension, without inflicting injury upon the ankle. We believe we are acquainted with, and have, at one time or another, tested most, or all of the various appliances recommended for the purpose of making extension in such a manner as to avoid unequal and injurious pressure upon the foot and ankle. Yet, we have, every now and then, produced vesications, ulcerations, and sloughings, in spite of all our care.

The three cases of fractured femur which follow, will illustrate what we hope to find a real improvement in this part of the treatment, and which we describe as follows:

The ankle, foot, and lower part of the leg is first completely swathed in thick sheets of carded cotton; over this, commencing at the toes, and extending to the knee, a starch bandage is carried; the whole limb being covered by, at least two turns of the roller, and the ankle and top of the foot with six or eight turns. After four or five turns are made about the ankle, a piece of binder's board may be laid along the inner and outer malleolus, to render this part of the gaiter more firm and unyielding. A firm piece of cotton roller must also be laid along the leg, on each side, and allowed to extend half a yard beyond the bottom of the foot, to be used as the extending band. This is secured by the remaining turns of the roller. The limb is now to be suspended slightly above the bed, to allow the air to approach it freely. If the weather is cold, hot bricks may be laid beside it, until it is perfectly dry. We have thus a gaiter which will not excoriate the limb under any reasonable amount of extension, and which need not be removed until the expiration of four or six weeks.

Another excellence which is claimed for this bandage is, that, unlike the the ordinary dry roller, applied to the leg and foot for the purpose of preventing oedema, it is not liable to become loosened or displaced.

Finally, we enumerate, briefly, the advantages which we propose in this new *appareil*:—

*Economy; facility of adaption; softness of the surface applied to the skin; firmness of the exterior, and the application of the extending forces to a variety of surfaces, viz., to the heel, instep, ankle, and above the calf of the leg. These circumstances greatly diminishing the danger of excoriations, &c.; permanence; support of the limb, and prevention of congestions, without the necessity of frequent readjustments.*

CASE 2. *Fracture of Femur.* James Brian, aet. 8 years, fractured his femur at the upper end of the lower third, Jan. 20, 1849. Fracture simple. Entered Hospital the next day. Jan. 23, we applied the starch bandage to his foot and leg. Jan. 28, seven days after the occurrence of the fracture, extension and counter-extension were made with a straight splint, and lateral splints were also applied. This treatment was continued until the 17th of Feb., a period of twenty days, and extending to twenty-seven days after the fracture; during which time, the little patient made no complaint, but was always cheerful and happy. On the 17th of February, the bandages and splints were removed. The bone had united with proper length and direction. There was not even discoloration, or tenderness, about the ankle or leg.

Case 3. John Gorty, aet. 13 years, fractured his femur, in middle third, Feb. 21, 1849. On the same day, we applied the starch bandage, from the toes to the knee Feb. 24, the bandages being dry, the straight splint was adjusted, and extension made. Lateral dressing were also applied. March 14, the fracture appeared firm. March 33, the bandages and splints were removed, and the leg was found sound and perfect. No excoriations had occurred on the foot or leg.

Case 4. G. W. Heyward, aet. 43 years, broke his left femur through the middle third, June 21, 1849. Same day we applied the starch bandage to his leg and foot, and seven days later we commenced the extension with the straight splint. The usual lateral splints were applied to the thigh. Starch was also used freely in the dressings about this part of the limb, in the application of the Scultetus bandage, and the padded splints, laid upon this bandage, were also covered over with the same. The effect of this has been, that the bandages held firmly to the limb, the splints to the bandage, and the roller to the splints, and the dressings did not require readjustment for three weeks.

Three weeks after the fracture occurred, the dressings and extending apparatus were all removed. Bone united, shortened half an inch, straight. No ulcerations of heel or instep.

Limb redressed with double inclined plane.

Case. 6. *Fracture of tibia and fibula; both legs.* Michael Carmody, aet. 19 years. May 9, 1849, a heavy bar of iron fell across his legs, breaking the right tibia and fibula, four inches above the ankle, and again near the middle. Both fractures of the tibia, oblique. The left tibia and fibula were slso broken, just above the ankle. Michael was received at the Hospital on the same day, and both legs were at once dressed with dry rollers, and supported with splints and pillows.

*May* 16, seven days after the fractures, the swelling of the limbs being considerably reduced, the starch bandage of M. Suetin was applied to each leg.

*May* 18, he was allowed to sit up in bed or in a chair. May 29, the swelling having still more subsided, the bandages were found slightly loosened. They were laid open and made smaller by cutting out a small strip, from the knee to the toes, and afterwards readjusted and secured by a dry roller.

*June* 13, five weeks after the fracture, the dressings were removed, and the limbs were sound and perfect, except that considerable stiffness existed in the left ankle joint.

In our private practice, we now resort to the starch or paste bandage af M. Suetin, in a considerable proportion of simple fractures of the arm, or forearm, and of the leg. The following will best illustrate its advantages, when the case and period for its application is properly chosen.

Case 6. *Fracture of tibia and fibula.* Wm. McCormick, aet. 19 years. In May, 1849, he broke both bones of the leg, near the middle. The fracture of the tibia was nearly transverse, and not much displaced. We saw the limb soon after the accident, and it was not swollen. The paste bandage was immediately applied, and on the third day after, he walked two miles by the aid of crutches, and during the whole treatment, four weeks, he was not confined to his bed one whole day. At the expiration of this period, the dressings were still snug, and the fracture had united, but with considerable callus, without bend or shortening.

Case 7. *Fracture of humerus, radius, and ulna.* Matthew Rigany, aet. 23 years. Admitted to the Hospital, May 7, 1849. The accident had occurred on the 3d, by having his arm caught in the crank of a hand-car when under full speed.

A broad *Gutta Percha* splint was applied to the palmar surface of the arm, forearm and hand, and a broad splint to the dorsal surface of the forearm, and smaller splints were laid around the humerus, and the whole secured by a roller.

On the 6th of June, the starch bandage was substituted, the swelling having now sufficiently subsided. On the 16th, the starch bandage was removed, and the patient dismissed, cured. There was no shortening of either arm or forearm; but the arm was slightly bent at the seat of fracture.

We would call the attention of our readers again, to the value of *Gutta Percha* as a splint. It may be obtained in Buffalo, at the store of Pratt & Letchworth, 165 Main st., of every degree of thickness, from the sixteenth to the quarter of an inch. The thick sheets should generally be preferred. These latter are sold at one dollar for a square foot. By immersing the splint in water near the boiling point, it is rendered perfectly flexible, and can be made to conform exactly to the shape of the limb. When cool, it becomes as firm, almost, as a board. It is not, however, affected sufficiently to impair its utility, by the temperature of the body, or by the vicissitudes of the air.

Gutta Percha not only becomes flexible by heat, but also plastic, and at length it melts like wax, and may be pressed into any form, or made to unite and consolidate ; yet its firmness is never by these processes impaired, when it again becomes cool. When melted or much heated, the surface should be slightly covered with oil, bofore it is applied to the body, or it will adhere. It is for fractures of the leg, arm, forearm, and inferior maxilla, that it is especially applicable. In the latter case, in addition to its service as an outside splint, it can be employed in another way, namely, as an *inside splint*, between the teeth, or for the purpose of keeping the jaws sufficiently asunder to admit of the introduction of nourishment. A piece of the proper size and shape is made soft, and then pressed between the teeth on each side, and now the fragments of the inferior maxilla are brought up until they are in place, and the jaw is sufficiently closed. In a few minutes the Gutta Percha is hard, and the crowns of the teeth are so imbedded in it, that it cannot become displaced. It is, when the teeth are well settled into it, a true *internal splint*, as well as wedge, and is incomparably superior to the wedge of wood or cork, heretofore employed. It is fixed, clean, smooth and unirritating to the tongue and mouth, and not so hard as to be painful to the teeth.

Case 8. *Fracture of radius and ulna.* Samuel Duckett, aet. 14 years. Admittted, Jan. 28, 1849. Four weeks since, he broke his left radius, near the wrist. An empiric said it was not broken. The deformity, at this point, is now very manifest, the hand inclining to the radial side of the arm, and the lower extremity of the ulna making an extraordinary projection to the ulnar side. Power of supination has not existed since the accident occurred.

Two days before his admission, (Jan. 26,) he fell again, and struck upon the elbow of the same arm. On the 28th, no crepitus could be discovered, and he could nearly straighten his arm. Jan 30, we discovered, when the olecranon was seized and attempted to be moved, a distinct crepitus. Until now, we did not discover that this was a fracture of the olecranon process, with very slight displacement.

The arm was extended, and a straight Gutta Percha splint applied. This, after four or five days, was daily changed; the splint being sometimes straight at the elbow joint, and at others bent at a more or less obtuse angle. On the 17th of Feb., twenty two days after the accident, the splints were removed, and the fracture was found united by bone, and the motions of the joint unimpaired.

Case 9. *Fracture of radius and ulna.* Mark Calligan, aet. 40 years. Fractured the bones of his forearm, two inches above the wrist, April 2, 1849. Admitted, April 4. We applied the Gutta Percha splint, on palmar and dorsal surface of forearm ; the palmar splint was made, also, to extend up the arm a short distance, to prevent the bandages from pressing upon the arm in front of the elbow. Twenty-one days after the fracture, the bones had not united. The splints were reapplied more firmly. Fourteen days later, the bones were firm, and the splints were removed. The cure was perfect.

